# Definitive Radiotherapy for Unresectable Unicentric Mediastinal Castleman Disease: A Case Report

**DOI:** 10.7759/cureus.110485

**Published:** 2026-06-08

**Authors:** Imane El Kachtouhi Aarim, Wafaa Merssetti, Siham Belkadi, Safae Regragui, Nabila Sellal

**Affiliations:** 1 Department of Radiation Oncology, Mohammed VI University Hospital Center, Tangier, MAR; 2 Department of Hematology, Mohammed VI University Hospital Center, Tangier, MAR

**Keywords:** case report, castleman disease, hyaline vascular subtype, mediastinal mass, radiation therapy, unicentric castleman disease, unresectable tumor, vmat

## Abstract

Castleman disease is a rare nonclonal lymphoproliferative disorder with heterogeneous clinical presentations and therapeutic approaches. Surgery remains the standard treatment for unicentric Castleman disease, while radiotherapy may represent an alternative option in unresectable cases.

We report the case of a 17-year-old female who presented with exertional dyspnea related to a right hilar mediastinal mass. Chest computed tomography revealed a well-circumscribed lesion closely related to adjacent vascular and bronchial structures. Histopathological examination demonstrated abnormal lymphoid follicles with atrophic germinal centers producing the characteristic “onion-skin” appearance. Immunohistochemical analysis showed a CD3−, CD20+, CD21+, Bcl2−, and CD5− profile consistent with the hyaline vascular subtype of unicentric Castleman disease. Positron emission tomography/computed tomography demonstrated localized hypermetabolic mediastinal lymphadenopathy without distant involvement. Due to close vascular relationships and high hemorrhagic risk, the lesion was considered unresectable after multidisciplinary discussion.

The patient underwent definitive volumetric modulated arc therapy (VMAT) delivering 39.6 Gy in 22 fractions. Treatment was well tolerated without significant acute or late toxicity. After 13 months of follow-up, clinical and radiological evaluation demonstrated a favorable response, with approximately 50% tumor reduction according to RECIST v1.1 criteria, improvement in dyspnea from New York Heart Association (NYHA) class III to class II, and normalization of pulmonary function tests.

This case highlights the potential role of definitive radiotherapy as an effective and well-tolerated therapeutic alternative in unresectable unicentric mediastinal Castleman disease.

## Introduction

Castleman disease (CD) is a rare nonclonal lymphoproliferative disorder characterized by marked etiological, clinical, therapeutic, and prognostic heterogeneity. It was first described in the 1950s by Benjamin Castleman as localized mediastinal lymph node hyperplasia associated with follicular and vascular proliferation with endothelial hyperplasia [1].

Over time, advances in pathology and molecular biology have led to the distinction of two main clinical forms: unicentric Castleman disease (UCD) and multicentric Castleman disease (MCD), the latter being either idiopathic (iMCD) or associated with human herpesvirus 8 (HHV-8) infection [2]. UCD generally presents as a localized mass with favorable outcomes, whereas MCD is characterized by diffuse lymph node involvement, systemic manifestations, and a more aggressive clinical course [2]. Histopathologically, three variants have been described: hyaline vascular, plasmacytic, and mixed [3].

Due to the rarity of this condition, no universal therapeutic consensus exists. Management mainly depends on the clinical form. In multicentric disease, treatment primarily relies on systemic approaches, including immunotherapy, corticosteroids, and/or chemotherapy [4]. In unicentric disease, surgery remains the standard treatment, providing excellent outcomes [5]. Radiotherapy represents a valuable therapeutic alternative in cases of unresectable disease or surgical contraindication [6].

We report the case of unresectable unicentric mediastinal CD treated with definitive radiotherapy.

## Case presentation

A 17-year-old female patient with no significant medical history presented with exertional dyspnea evolving over two months, classified as New York Heart Association (NYHA) class III. She denied constitutional symptoms, including fever, weight loss, or night sweats. Physical examination revealed no palpable peripheral lymphadenopathy.

Chest computed tomography (CT) revealed a well-circumscribed right hilar mediastinal mass measuring 52 × 51 × 48 mm. The lesion demonstrated close relationships with adjacent vascular and bronchial structures: it was in intimate contact with the superior vena cava, causing partial compressive, partially encased the right pulmonary artery, and was in contact with the right main bronchus, the upper and middle lobar bronchi, and the right atrium (Figure 1).

**Figure 1:**
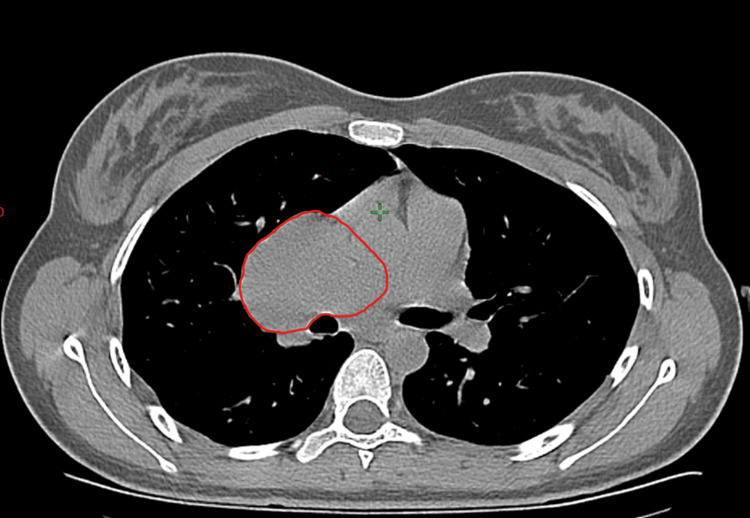
Initial thoracic imaging. Axial chest CT scan showing a right hilar mediastinal mass in close contact with adjacent vascular and bronchial structures.

A lymph node biopsy demonstrated altered nodal architecture with abnormal lymphoid follicles containing reduced and atrophic germinal centers, producing the characteristic “onion-skin” appearance.

Immunohistochemical analysis showed a CD3−, CD20+, CD21+, CD23+, Bcl2−, and CD5− profile. The overall morphologic and immunohistochemical findings were consistent with the hyaline vascular subtype of CD.

As part of the staging workup, positron emission tomography (PET)/CT revealed a cluster of hypermetabolic lymphadenopathies involving the right hilar region, Barety’s lodge, and the subcarinal region, with a maximum standardized uptake value (SUVmax) of 3.65 and no evidence of distant disease. Laboratory investigations showed normal lactate dehydrogenase (LDH) levels at 140 IU/L (reference range: 135-245). C-reactive protein (CRP), erythrocyte sedimentation rate, and serum albumin were within normal ranges, with no detectable monoclonal abnormalities on protein immunofixation studies. HIV-1, HIV-2, HHV-8, Epstein-Barr virus (EBV), cytomegalovirus (CMV), hepatitis B, and hepatitis C serologies were negative. Pulmonary function tests demonstrated an obstructive ventilatory disorder without significant reversibility after bronchodilator testing.

Based on the clinical, radiological, histopathological, and immunohistochemical findings, a diagnosis of unicentric mediastinal hyaline vascular CD was established.

Following multidisciplinary team discussion, the tumor was considered unresectable because of its close vascular relationships and high hemorrhagic risk. Definitive radiotherapy was therefore selected as the treatment approach.

The patient received intensity-modulated conformal radiotherapy using volumetric modulated arc therapy (VMAT) (Figure 2), delivering a total dose of 39.6 Gy in 22 fractions of 1.8 Gy.

**Figure 2:**
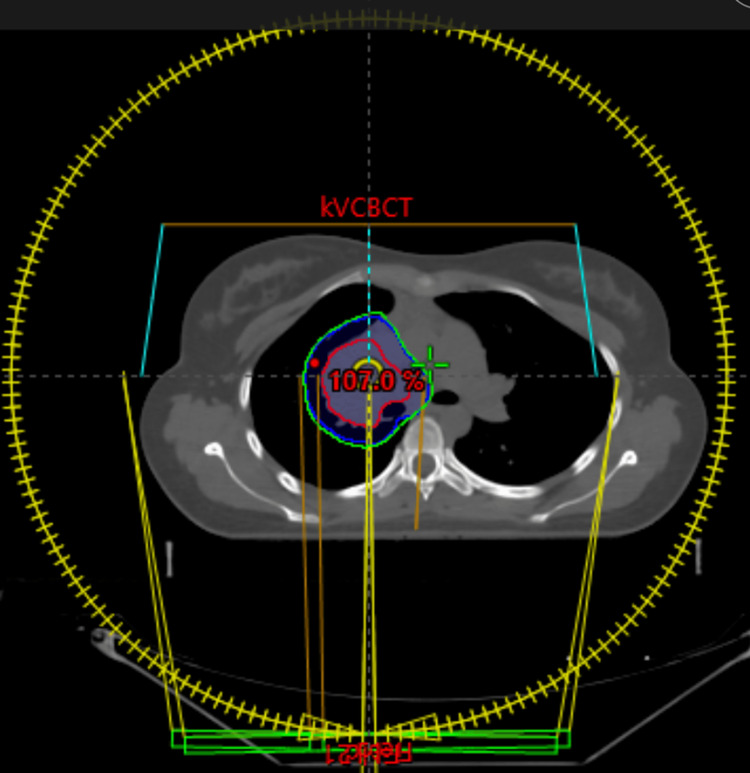
Axial radiotherapy planning image using the volumetric modulated arc therapy (VMAT) technique.

Dosimetric analysis demonstrated excellent planning target volume (PTV) coverage, with D98, D50, and D2 corresponding to 97.6%, 101%, and 106% of the prescribed dose, respectively, indicating homogeneous (HI = 0.089) and conformal (CI ≈ 1) dose distribution. Organs-at-risk constraints were globally respected, with a lung V20 of 26.77%, mean heart dose of 12.1 Gy, mean esophageal dose of 20.8 Gy, and maximum spinal cord dose of 7.9 Gy.

After a mean follow-up of 13 months, clinical and radiological evaluation demonstrated a favorable response. Follow-up chest CT performed at six months showed significant tumor shrinkage, with the mass measuring 26 × 28 × 30 mm (Figure 3), corresponding to approximately 50% reduction in the largest diameter and consistent with a partial response according to RECIST v1.1 criteria.

**Figure 3:**
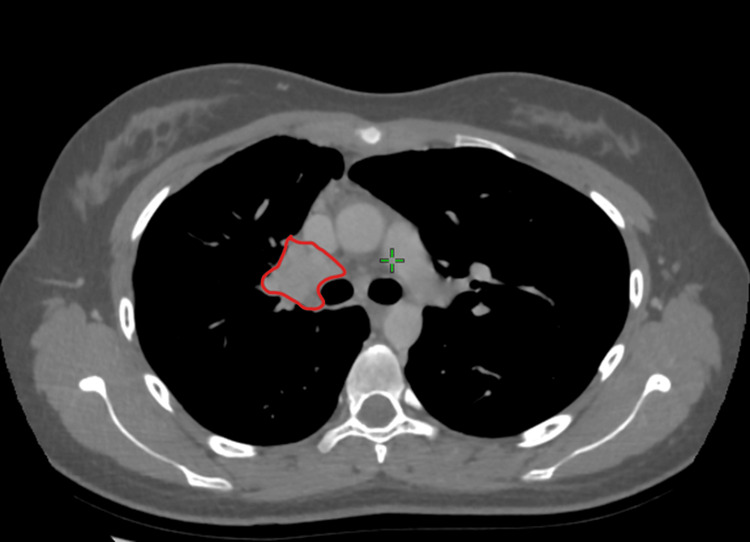
Axial follow-up chest CT scan at six months demonstrating marked reduction in the right hilar mediastinal mass.

Clinically, marked improvement was observed, with NYHA status improving from class III to class II. Follow-up pulmonary function tests showed normalization of ventilatory parameters. No acute or late grade ≥ 2 toxicities according to CTCAE v5.0 were observed.

## Discussion

The incidence of CD is estimated at 21-25 cases per million person-years, corresponding to approximately 4,300-5,200 new cases annually in the United States [7]. UCD can occur at any age, with a median age around 34 years and a female predominance of approximately 60% [8,9]. Our patient fits the epidemiological profile described in the literature, although her age was younger than the reported median.

Clinically, UCD usually presents as a localized lymph node mass, often asymptomatic and incidentally discovered, with symptoms mainly related to compression of adjacent structures. The mediastinum remains the most commonly involved site [10]. In our case, exertional dyspnea was explained by the hilar mediastinal location and the compressive effect exerted on the superior vena cava, right pulmonary artery, and bronchial structures.

Histopathologically, the hyaline vascular subtype is the most frequent form. It is characterized by atrophic and hyalinized follicles surrounded by concentric mantle zone hyperplasia producing the characteristic “onion-skin” appearance, associated with prominent vascular proliferation. Immunohistochemically, the typical profile includes CD20+, CD21+, CD23+, BCL2−, and CD3− expression without evidence of clonal proliferation. The histological and immunohistochemical profile observed in our patient was consistent with this subtype [11]. 

The diagnosis of UCD relies on a multidisciplinary approach integrating clinical, radiological, histopathological, and immunohistochemical findings [12]. The differential diagnosis is broad and includes several lymphoproliferative, infectious, inflammatory, and neoplastic disorders that may share overlapping histopathological features. The main differential diagnoses include lymphomas, particularly Hodgkin lymphoma, certain variants of follicular lymphoma, angioimmunoblastic T-cell lymphoma, thymomas, and reactive lymphoid proliferations. Autoimmune diseases and viral infections may also mimic CD, particularly infections related to HIV, HHV-8, EBV, and CMV [12].

Therefore, the diagnostic workup should include a comprehensive clinical evaluation for constitutional or inflammatory symptoms, imaging studies to assess lymph node extension, and laboratory investigations including CRP, erythrocyte sedimentation rate, serum albumin, protein immunofixation studies, and viral serologies [12]. In our case, HIV, HHV-8, EBV, CMV, hepatitis B, and hepatitis C serologies were negative. In addition, the absence of biological inflammatory syndrome, with normal CRP, erythrocyte sedimentation rate, and serum albumin, as well as the absence of detectable monoclonal abnormalities on protein immunofixation, further supported the diagnosis of localized unicentric disease without systemic inflammatory syndrome.

Surgery remains the standard treatment for UCD and provides excellent local control rates. A systematic review reported five-year overall survival rates exceeding 95% after complete resection, with recurrence rates as low as 5% [9].

However, certain locations, particularly mediastinal lesions, may render surgical resection difficult or even impossible. In such situations, several therapeutic options may be considered, including debulking surgery, preoperative embolization, cytoreductive medical therapies (corticosteroids, rituximab, anti-IL-6 antibodies), and radiotherapy [13]. According to current recommendations [14], radiotherapy represents a valid therapeutic alternative for unresectable unicentric disease. Initially considered poorly effective [3], more recent series have demonstrated significant response rates, supporting its use in this setting [15].

Available literature suggests that doses ranging from 30 to 45 Gy provide satisfactory tumor control in unresectable disease. In the series reported by Chronowski G et al., four patients were treated using two fractionation schedules (40 Gy in 2 Gy fractions and 39.6 Gy in 1.8 Gy fractions), achieving complete radiological remission in three patients, with a median follow-up of 30 months [15]. Nevertheless, the level of evidence remains limited due to the rarity of the disease and the predominance of retrospective studies.

In our case, definitive radiotherapy achieved approximately 50% tumor reduction associated with marked clinical improvement after 13 months of follow-up. However, long-term follow-up remains essential to assess response durability and detect potential late complications or recurrence.

## Conclusions

Surgery remains the standard treatment for UCD. However, radiotherapy represents an effective alternative in unresectable cases, providing durable tumor control with good tolerance.

Our case highlights the role of radiotherapy as a relevant therapeutic option when surgery is not feasible. It also underlines the importance of structuring the management of rare diseases through the establishment of national registries and referral networks to expert centers in order to optimize patient care and harmonize therapeutic strategies.

## References

[1] Castleman B, Iverson L, Menendez VP (1956). Localized mediastinal lymphnode hyperplasia resembling thymoma. Cancer.

[2] Dispenzieri A, Fajgenbaum DC (2020). Overview of Castleman disease. Blood.

[3] Keller AR, Hochholzer L, Castleman B (1972). Hyaline-vascular and plasma-cell types of giant lymph node hyperplasia. Cancer.

[4] van Rhee F, Voorhees P, Dispenzieri A (2018). International, evidence-based consensus treatment guidelines for idiopathic multicentric Castleman disease. Blood.

[5] Shahidi H, Myers JL, Kvale PA (1995). Castleman's disease. Mayo Clin Proc.

[6] Li YM, Liu PH, Zhang YH (2011). Radiotherapy of unicentric mediastinal Castleman's disease. Chin J Cancer.

[7] Munshi N, Mehra M, van de Velde H, Desai A, Potluri R, Vermeulen J (2015). Use of a claims database to characterize and estimate the incidence rate for Castleman disease. Leuk Lymphoma.

[8] Ye B, Gao SG, Li W (2010). A retrospective study of unicentric and multicentric Castleman's disease: a report of 52 patients. Med Oncol.

[9] Talat N, Belgaumkar AP, Schulte KM (2012). Surgery in Castleman's disease: a systematic review of 404 published cases. Ann Surg.

[10] Legras A, Tallet A, Didelot A (2018). Clinical and molecular characteristics of unicentric mediastinal Castleman disease. J Thorac Dis.

[11] Wu D, Lim MS, Jaffe ES (2018). Pathology of Castleman disease. Hematol Oncol Clin North Am.

[12] van Rhee F, Oksenhendler E, Srkalovic G (2020). International evidence-based consensus diagnostic and treatment guidelines for unicentric Castleman disease. Blood Adv.

[13] (2019). Protocole National de Diagnostic et de Soins (PNDS): Maladie de Castleman. https://www.has-sante.fr/upload/docs/application/pdf/2019-11/pnds_castleman_2019.pdf.

[14] (2026). NCCN clinical practice guidelines in oncology: Castleman disease. Version 1.2025. https://www.nccn.org/professionals/physician_gls/pdf/castleman.pdf.

[15] Chronowski GM, Ha CS, Wilder RB, Cabanillas F, Manning J, Cox JD (2001). Treatment of unicentric and multicentric Castleman disease and the role of radiotherapy. Cancer.

